# Prevalence of Anemia in Adolescent Girls attending Specific Schools of Kavrepalanchok, Nepal

**DOI:** 10.31729/jnma.6330

**Published:** 2021-03-31

**Authors:** Lee Budhathoki, Bikal Shrestha, Naveen Phuyal, Lochana Shrestha

**Affiliations:** 1 Department of Community Medicine, Nepalese Army Institute of Health Sciences, Sanobharyang, Kathmandu, Nepal

**Keywords:** *adolescent*, *anemia*, *Nepal*

## Abstract

**Introduction:**

Adolescents comprise individuals in the age group 10 to 19 who are undergoing a period of gradual transition of growth and development. Adolescent girls are at high risk of anemia and malnutrition. This research has been aimed to find the prevalence of anemia in adolescent girls attending specific schools of Panchkhal municipality in Kavrepalanchowk in Nepal.

**Methods:**

A descriptive cross-sectional study was conducted in one government and one private school of Panchkhal Municipality of Kavre district in adolescent girls of age 10 to 19 from December 2017 to April 2018 attending grade five till ten in the field practice area of a medical college. After ethical approval, anthropometric measurements of height, weight, and blood were taken for hemoglobin estimation. Descriptive analysis was done in Microsoft excel 2016.

**Results:**

Out of 95 adolescent girls, fourteen (14.74%) adolescent girls were anemic. The mean age was 13.35±1.629 years, mean height and weight were 1.58m and 44.11kg, respectively.

**Conclusions:**

This study shows a lower prevalence of anemia than reported by national data, but it is higher than that reported by developed countries. Anemia and undernutrition have long terms detrimental effects on adolescent girls, so health education and prevention is necessary.

## INTRODUCTION

Individuals in the age group of 10 to 19 years are called Adolescents.^1^ Out of the estimated nearly 1.2 billion adolescents, 350 million live in South East Asia.^1,2^ In Nepal, almost one-fourth of the population, are adolescents who are undergoing a period of gradual transition of growth and development with unique physical, mental and emotional challenges.^3^ Adolescent girls are at high risk for anemia and malnutrition.^4^ One-fourth to half of the adolescent girls in South East Asia are anemic, and up to one in six are too thin.^4,5^

Inadequate nutrition during adolescence can have serious consequences throughout the reproductive years of life and beyond, increasing morbidity and mortality. Many of these girls can be reached through schools.

The present study was undertaken to find the prevalence of anemia among adolescent girls in adolescent girls attending specific schools of Panchkhal Municipality of Kavre district in the community field posting area of a medical college.

## METHODS

This is a descriptive cross-sectional study conducted in schools of Panchkhal Municipality of Kavre district in the first-year community field practice area of College of Medicine, Nepalese Army Institute of Health Sciences. The study proposal was given ethical approval from the Institutional Review Committee of the institution which was sent to the Nepal Health Research Council (NHRC) for a study grant that was approved. Written informed consent from the Principals and verbal consent from the students was taken, and data were collected from December 2017 to April 2018.

All schools in Panchkhal municipality in the field practice area were listed. There were 11 schools, out of which five were excluded as they had students till grade eight. Out of the remaining six, four were run by the government, and two were run by the private sector. One school of private and one government was selected by lottery method. Sample size was then calculated by taking data for the prevalence of anemia in rural field practice area medical college of India with finite correction by using formula.

$$\begin{array}{ll} \text{n} & ={\text{Z}}^{\text{2}}\times \text{p}\times \text{q}/{\text{e}}^{\text{2}}\\ & ={\left(1.96\right)}^{2}\times 0.41\times 0.59/{\left(0.08\right)}^{\text{2}}\\ & =145.20 \end{array}$$

Where,

n = number of sample size
Z = 1.96 at 95% confidence interval
p = prevalence, 41%^6^
q = 1-p
= 1-0.41
= 0.59
e = margin of error, 8%

For finite population calculation, the total number of female students from grade five till ten in the age group of 10-19 years were listed, which came out to be 224 (N).

Adjusted sample size = n / [1+{(n-1)/N}]

    = 88.33

Adding a non-response rate of 5%, the adjusted minimum sample size was calculated as 94. A total of 95 adolescent girls fulfilling the inclusion and exclusion criteria were included in the study. Students were randomly selected till the sample size was met. Two students did not give an consent for hemoglobin collection, so their data were also excluded from anthropometric measurement.

Information about the socio-demographic characteristics was recorded in the proforma. Drawing of blood for hemoglobin estimation was done. Anemia is said to be present when the hemoglobin level in the blood is below the lower extreme of the normal range for the age and sex of the individual. According to the WHO criteria, the cut off level of the hemoglobin concentration in blood for the diagnosis of anemia is less than 11gm/dl for pregnant women and for children who are aged between 6 months and 6 years, less than 12 gm/dl for non-pregnant women and children who are aged 6-14 years old, and less than 13 gm/dl for adult males.^7^ The adolescent girls were thus classified as anemic if Hb was less than 12 gm%. Hemoglobin estimation was done by collecting 2 ml of venous blood, drawn by venipuncture from an antecubital vein under aseptic precautions, collected in EDTA (Ethylenediaminetetraacetic acid) vacutainers and analyzed by using the Cynmeth method. The final disposal of the sample was done by treating it with Sodium hypochlorite solution. Syringes and vacutainers were autoclaved before discarding. Age was recorded to the nearest completed years and was determined from the register of the school. Data was entered and analyzed in Microsoft excel 2016.

## RESULTS

Ninety-five adolescent girls of the age group 10 to 18 years with a mean age of 13.35±1.629 years from grade five till ten were taken in the study. A majority of participants, 69 (72.63%), were more than 14 years of age. 89 (93.7%) of the study participants followed the Hindu religion, followed by Christianity 4 (4.2%) and Buddhism 2 (2.1%). 46 (48.42%) participants in the study group belonged to Janjati, followed by Brahmin 27 (28.42%), Chettri 19 (20%) and Dalit 3 (3.15%), respectively. Sixty-six (69.47%) adolescent girls were from a government school, and 29 (30.53%) were from private-owned schools. Eighty-four (88.42%) of participants consumed the non-vegetarian diet.

**Figure 1.**
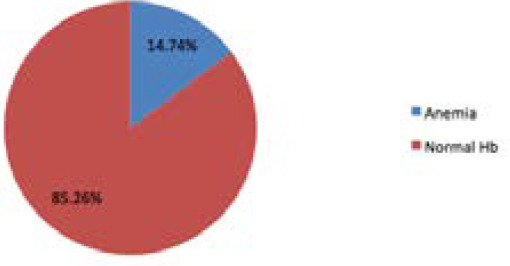
Prevalence of anemia in study participants.

The mean and standard deviation of various parameters collected during the study (Table 1). Fourteen (14.74%) adolescent girls had anemia at the time of the study (Figure 1).

**Table 1:** Mean and standard deviation of various study variables.

	Age (years)	Hb (gm/dl)	Ht (m)	Weight (kg)
Mean ± SD	13.35 ± 1.629	13.28 ± 1.53	1.48 ±.058	44.11 ± 8.58

## DISCUSSION

Our study reports the prevalence of anemia in adolescent school-going girls as 14.74%. As per the Nepal National micronutrient status survey, the prevalence of anemia in 10-19 aged adolescent girls was 20.5%.^9^ A study by Limbu et al. in Dharan, Nepal, reports the prevalence of 33.33% in school-going adolescent girls.^10^ Chalise et al. and Hamal et al. give a prevalence of 38% and 42.5% respectively in adolescent girls of Nepal.^11,12^ In India, a survey by the Ministry of Health and Family Welfare, the National Family Health Survey (NFHS4), reports the prevalence of anemia in women of age 15-49 to be 53%.^13^ Various other studies done in India in adolescent girls report prevalence from 35.1% to 98%.^14,15^ All these studies thus show a higher prevalence of anemia than that reported in our study.

However, studies done in more developed countries have reported a lower percentage of anemia. In a study done in Kuwait by Shaban et al., 10.96% of adolescent girls had anemia.^16^ A similar report was given by Looker et al. who reports 9% of adolescent girls of the United States of America being anemic.^17^ Balci et al. report 8.3% of adolescent girls with anemia in Turkey,^18^ while in Spain, 1.2% of adolescents had iron deficiency anemia.^19^

The lower percentage of anemia among our study population compared to national data and other studies done in developing countries could be attributed to the participants being selected from the community field posting area of a medical college where regular health education on proper nutrition intake and anemia is given. The majority of our participants (88.42%) consumed a non-vegetarian diet, which could be the cause of low prevalence as studies suggest that the strongest predictor of anemia in adolescent girls was the vegetarian diet.^20^ The percentage is higher than that of developed countries due to these countries having better nutrition and socio-economic status, as various studies show that anemia is significantly associated with better nutrition, socio-economic status, and educational status.^15,21^

The limitation of the study is that it did not consider all the factors responsible for the cause of anemia. The study was also conducted in two schools in a specific area, so the study results cannot be generalized.

## CONCLUSIONS

Prevalence of anemia was found to be lower compared to national data. However, adolescence is a critical period of life with various physical and physiological changes occurring, so further reduction of lower hemoglobin level is required at this period as it has long-term effects on health. Regular supplementation of iron and folic acid and investigation to find the cause of anemia needs to be done. Schools should start health education on subjects related to nutrition as anemia is detrimental to health.
